# Effect of aqueous extract of *Tinospora cordifolia *on functions of peritoneal macrophages isolated from CCl_4 _intoxicated male albino mice

**DOI:** 10.1186/1472-6882-11-102

**Published:** 2011-10-28

**Authors:** Mahuya Sengupta, Gauri D Sharma, Biswajit Chakraborty

**Affiliations:** 1 Department of Biotechnology, Assam University, Silchar-788 011, Assam; India; 2 Department of Life Science, Assam University, Silchar-788 011, Assam; India

## Abstract

**Background:**

The current practice of ingesting phytochemicals for supporting the immune system or fighting infections is based on centuries-old tradition. Macrophages are involved at all the stages of an immune response. The present study focuses on the immunostimulant properties of *Tinospora cordifolia *extract that are exerted on circulating macrophages isolated from CCl_4 _(0.5 ml/kg body weight) intoxicated male albino mice.

**Methods:**

Apart from damaging the liver system, carbon tetrachloride also inhibits macrophage functions thus, creating an immunocompromised state, as is evident from the present study. Such cell functions include cell morphology, adhesion property, phagocytosis, enzyme release (myeloperoxidase or MPO), nitric oxide (NO) release, intracellular survival of ingested bacteria and DNA fragmentation in peritoneal macrophages isolated from these immunocompromised mice. *T. cordifolia *extract was tested for acute toxicity at the given dose (150 mg/kg body weight) by lactate dehydrogenase (LDH) assay.

**Results:**

The number of morphologically altered macrophages was increased in mice exposed to CCl_4_. Administration of CCl_4 _(i.p.) also reduced the phagocytosis, cell adhesion, MPO release, NO release properties of circulating macrophages of mice. The DNA fragmentation of peritoneal macrophages was observed to be higher in CCl_4 _intoxicated mice. The bacterial killing capacity of peritoneal macrophages was also adversely affected by CCl_4. _However oral administration of aqueous fraction of *Tinospora cordifolia *stem parts at a dose of 40 mg/kg body weight (*in vivo*) in CCl_4 _exposed mice ameliorated the effect of CCl_4_, as the percentage of morphologically altered macrophages, phagocytosis activity, cell adhesion, MPO release, NO release, DNA fragmentation and intracellular killing capacity of CCl_4 _intoxicated peritoneal macrophages came closer to those of the control group. No acute toxicity was identified in oral administration of the aqueous extract of *Tinospora cordifolia *at a dose of 150 mg/kg body weight.

**Conclusion:**

From our findings it can be suggested that, polar fractions of *Tinospora cordifolia *stem parts contain major bioactive compounds, which directly act on peritoneal macrophages and have been found to boost the non-specific host defenses of the immune system. However, the molecular mechanism of this activity of *Tinospora cordifolia *on immune functions needs to be elucidated.

## Background

The immune system is increasingly found to be challenged by several chronic illnesses, for which allopathic medicine has provided limited tools of treatment and especially prevention. In that context, it appears worthwhile to target the immune system in order to modulate the risk of certain chronic illnesses. Meanwhile, natural health products (NHPs) are generating renewed interest, particularly in the prevention and treatment of several chronic diseases. Macrophages are armed with several mechanisms to destroy microorganisms within phagosomes, which include nutrient limitation, toxic oxygen and nitrogen intermediates, acidification of the compartment and fusion of phagosomes with lysosomes that are rich in hydrolytic enzymes. Herbal drugs are known to posses immunomodulatory properties and generally act by stimulating or suppressing both specific and non-specific immunity.

The herb *Tinospora cordifolia *(*T. cordifolia*, Menispermaceae) belongs to a group of medicinal plants that grows in the tropical and subtropical regions of India. It is a large glabrous climber with succulent corky stem, subdeltoid cordate leaves, branches sending down, and pendulous fleshy roots. The herb is extensively used in the Indian System of Medicine; the extract of different parts of the herb has found wide use in variety of diseases. It is known for its immunomodulatory, antioxidant, and antibacterial properties [1-3]. The novel (1,4)-alpha-D-glucan from *Tinospora cordifolia *activates the immune system through the activation of macrophages that occurs through TLR6 signaling, NF kappa B translocation and cytokine production[4]. The hydro-methanolic extract of *T. cordifolia *(stem) possesses antibacterial and immunomodulatory properties [5]. The water soluble fraction of *T. cordifolia *leaf fraction has immunostimulatory and disease resistance properties and has potential to be used as an immunoprophylactic agent [6]. Although various literatures suggested that *T. cordifolia *has immunomodulatory properties very few reporting was observed regarding the effect of *T. cordifolia *in relation to non specific host response.

It has been well reported that CCl_4 _causes liver damage; as such, this model of hepatotoxicity [7] has been widely used to study the protective effect of any exogenous drug in an experimental animal model. Hepatotoxicity and its allied symptoms of jaundice are routinely associated with immunosuppression and existence of an immunocompromised state that leads to opportunistic infections. In our study for inducing hepatotoxicity and associated immune dysfunction, carbon tetrachloride was administered at 0.5 ml/kg b.w. for 7 days. Animals were then fed an aqueous stem-extract of *T. cordifolia*. Our results provide evidence of the adverse effect of CCl_4 _on the liver functions [8] and associated immunosuppression. The objective of creating an immunocompromised state in mice having hepatic injury was to mimic such condition occurring naturally in pathological manifestation of jaundice.

In a previous study by Bishayi *et al *[9], it was reported that CCl_4 _- induced hepatotoxicity is ameliorated by *T. cordifolia *extract (100 mg/kg. body weight) as evident from the serum enzyme levels viz., SGOT, SGPT and alkaline phosphatase. However the mechanism of CCl_4_-induced immunotoxicity and the deletion of such immunosuppressive effects by *T. cordifolia *have been fully elucidated in the present study. Peritoneal macrophage morphology and cell functions like, cell adhesion, phagocytosis, myeloperoxidase (MPO) release, nitric oxide (NO) release and intracellular killing were studied in this present work. DNA fragmentation was estimated as an indicator of cell death. The present study was aimed at evaluating the immunomodulatory properties of *T. cordifolia *on function of peritoneal macrophages isolated from CCl_4 _intoxicated male albino mice.

## Methods

### Preparation of Extracts

#### Collection of plant

*T. cordifolia *samples were collected from niches of wild flora adjoining Assam University and neighboring areas of Silchar.

#### Grinding of the selected plant materials

After drying at 37°C for 72 h the plant material was ground into powder. Exposure to sunlight was avoided to prevent the loss of active components.

### Extraction of selected plant material powder by maceration method

One liter of double distilled water was mixed with 100 g of powdered *T. cordifolia *stem, filtered twice with Whatman no.1 and then with nitrocellulose membrane. The extracted liquid was subjected to water bath evaporation to remove the water. For water bath evaporation, liquid extract material was be placed into a beaker and subjected to water bath evaporation at 60°C temperature for 7-10 h daily for 2-3 days until a semisolid state of extracted liquid is obtained. The semisolid extract produced was kept in the deep freezer at -20°C overnight and then subjected to freeze drying. Extract obtained by this method was then weighed and stored at 22°C in desiccators until further use. The mice were fed with powdered plant material extract mixed with sterile tap water. Phytochemical screening of the aqueous extract of *T. cordifolia *was also carried out (Table 1).

**Table 1:** Phytochemical screening of aqueous extract of *T.cordifolia *stem parts

Alkaloids	Glycosides	Reducing sugar	Saponins	Tannins	Polyphenols
Present	Present	Present	Present	Present	absent

### Animals

Twenty Swiss male albino mice weighing approximately (20 ± 1.0 g) were taken and these mice were divided into four groups of five mice each. The first group was administered (i.p.) with 0.1 ml sterile isotonic saline and kept as control. The second group comprised of mice treated with CCl_4 _at a dose of 0.5 ml/kg b.w. (i.p.) administered in the last 7 days of the experiment. In the third group, the mice were orally administered with extract of *T. cordifolia *at a concentration of 40 mg/kg b.w. by feeding needle for 15 days. In the fourth group, the mice were administered with *T. cordifolia *extract (for 15 days, orally) and CCl_4 _(for last 7 days, i.p.). Animal experiments were in accordance with the instructions for the care and use provided by the institution at which the research was carried out. The study was approved by the Institutional Animal Ethics Committee (IAEC), Assam University, Silchar.

### Isolation of peritoneal macrophages

On day 13, all the mice were injected with 50 μl of 3% starch (i.p). After two days they were sacrificed by cervical dislocation. Following this 5 ml of ice-cold RPMI-1640 were injected (i.p) in all the dead mice and the injected area was lavaged softly. A small perforation was made to withdraw the RPMI-1640 containing peritoneal fluid. The process was repeated to accumulate the remaining fluid by aspirating and collecting in plastic centrifuge tubes. The samples were then centrifuged for 30 min at 3500 rpm. All the samples were then washed in RPMI-1640 twice and the pellet were collected separately and incubated for 2 h at 37°C. The supernatant was decanted and the adhered macrophages present in the micro centrifuge tubes were resuspended in 1 ml RPMI-1640. A portion of entire cell samples (100 μl) isolated from all the mice, were then aspirated and smeared on a glass slide and incubated for 1 h, fixed with 4.1% formaldehyde, kept for 30 min before staining with Giemsa and all these assay were performed separately for each group of mice. After sometime the slides were washed, air dried and observed under microscope. The other portions of cell samples were kept intact for further assays [10].

### Morphological alteration assay

A volume of 100 μl sample cells in HBSS-BSA from all mice was taken separately and fixed in an equal volume of 2.5% glutaraldehyde in HBSS (Hank's buffered salt solution). After 10 min, cells were centrifuged at 2000 rpm for 5 min. The supernatant was removed and the pellet was resuspended in HBSS. Smears of cells were drawn on glass slides, air dried, fixed in methanol and stained with Giemsa. Cells were observed under oil immersion microscope. Any cell having intensive rough surface was scored as polarized and this was expressed as a percentage of the total number of cells counted [11].

### *In vitro *cell adhesion assay

Cells were seeded separately for different groups in 96-well microtitre plates and allowed to adhere for different times (0, 20, 40 and 60 min). In time, wells were washed with HBSS, and then 100 μl of 0.5% crystal violet in 12% neutral formaldehyde, and 10% ethanol was added to each well and incubated for 4 h to fix and stain the cells. Wells were washed and air dried for 30 min crystal violet was extracted from the macrophage adhered in the wells by lysing with 0.1% SDS in HBSS. Absorbance was measured spectrophotometrically at 570 nm. Cell adhesion was measured at 0, 20, 40 and 60 min and expressed as increased absorbance at 570 nm [12].

### Phagocytosis assay

A volume of 100 μl of cells from all mice was allowed to adhere separately on glass slides whereas non adherent cells were washed out with DPBS (1X). To the glass slides containing adhered macrophages 10% heat killed *Staphylococcus aureus *was added and incubated for 3 h at 37°C which were then washed with DPBS (1X) and dried. The cells were at last fixed in 50% methanol, stained with Giemsa, observed under oil immersion microscope and counted for number of bacterial cells ingested [13].

### Myeloperoxidase release assay

A volume of 200 μl of cell suspension from different groups was taken into micro centrifuge tubes and stimulated with LPS (100 ng/ml) for 1 h at 37°C and centrifuged at 13000 rpm for 10 min. The supernatant thus obtained from different sets was recovered separately and kept at -20°C until further use. The cell free supernatant was used for assay of the partial MPO release for different groups. The pellet that recovered from all the four groups were lysed in 0.01% SDS and then centrifuged again; the supernatant was recovered as before for total MPO release assay. Subsequently 100 μl of cell free supernatant as well as from lysis of cells were reacted with 100 μl substrate buffer (orthophenylenediamine) and kept at 37°C for 20 min, then the reaction was stopped by adding 100 μl of 2(N) H_2_SO_4 _and absorbance was measured at 492 nm [14].

### Nitric oxide release assay

100 μl of macrophage cells were isolated from respective groups from 10^6 ^cells/ml dilution and then suspended in DPBS-BSA. The cells were than stimulated with LPS (100 ng/ml) for 1 h at 37°C [15].

### DNA fragmentation assay

The DPA reaction was performed according to the method described elsewhere [16]. Perchloric acid (0.5 M) was added to the pellets containing uncut DNA (resuspended in 200 μl of hypotonic lysis buffer) and to the other half of the supernatant containing DNA fragments. Then 2 volumes of a solution containing 0.088 M diphenylamine (DPA), 98% (vol/vol) glacial acetic acid, 1.5% (vol/vol) sulfuric acid, and a 0.5% (vol/vol) concentration of 1.6% acetaldehyde solution were added. The samples were stored at 4°C for 48 h. The colorimetric reaction was quantified spectrophotometrically at 575 nm. The percent fragmentation was calculated as the ratio of DNA in the supernatants to the total DNA [17].

### Intracellular killing assay

In this procedure, 100 μl of bacterial cells were incubated separately for all groups with macrophages in a total volume of 1 ml DPBS-BSA and kept in the shaker for 20 min at 37°C. Non ingested bacteria (*Staphylococcus aureus*) were then removed by differential centrifugation for 10 min at 10000 rpm at 4°C. Following that, two washes were given with ice-cold DPBS-BSA. The cells containing ingested bacteria is resuspended in DPBS-BSA containing 10% normal serum and incubated at 37°C for 25 min. At 0 min and after 15, 30 and 45 min time intervals 0.1 ml sample is removed each time and treated with gentamycin. The above content was then plated onto nutrient agar petri plate and the number of viable intracellular bacteria was determined by counting the individual colony formed [18].

### Statistical analysis

A one-tailed Student's t test as well as ANOVA was performed to compare the mean values of control and *T. cordifolia *extract group. The results are expressed as mean ± standard error mean and all the experiment was done in triplicates. (Note: P < 0.0001 as given as P = ****, P < 0.001 = P***, P < 0.01 = P** and P < 0.05 = P*)

## Result

### Effect of *Tinospora cordifolia *on morphology of peritoneal macrophages isolated from CCl_4 _intoxicated male albino mice

**Figure 1:**
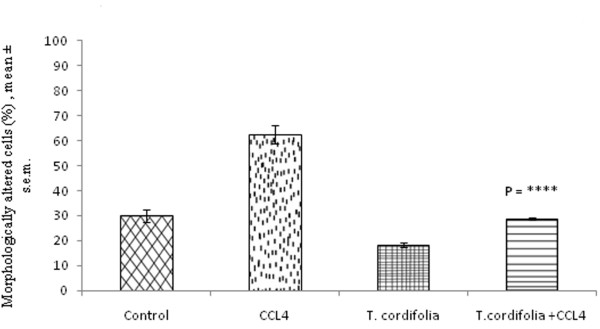
**Effect of *T. cordifolia *on morphology of peritoneal macrophages isolated from CCl_4 _intoxicated male albino mice**. The results were expressed as mean ± standard error mean.

Morphology of macrophage plays a prominent part of their function. To demonstrate the effect of both CCl_4 _and *T. cordifolia *on the number of differentiated macrophages, the macrophage cells of extensive rough surface or perfect spherical surface was measured and counted as morphologically altered macrophage. From this experiment, it was found that CCl_4 _treatment increases the number of altered macrophages from 30 ± 2.63% to 62.4 ± 3.54%. However the administration of *T. cordifolia *in CCl_4 _intoxicated mice, the number of altered macrophages became almost normal level of 28.68 ± 0.52%. Administration of *T. cordifolia *in control mice also showed some significant reduction of altered macrophages up to a level of 18.32 ± 1.02% (Figure 1; P = ****).

### Effect of *Tinospora cordifolia *extract on the adhesion property of CCl_4 _intoxicated mice peritoneal macrophages

**Figure 2:**
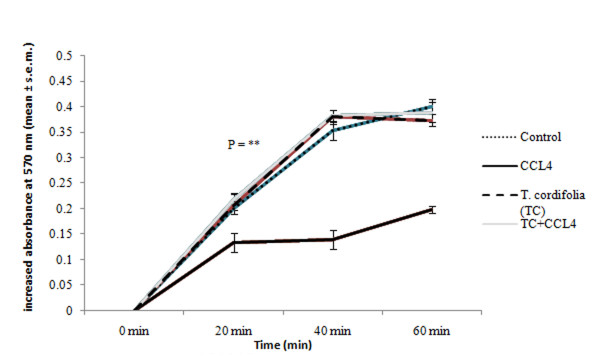
**Effect of *T. cordifolia *on cell adhesion properties of peritoneal macrophages isolated from CCl_4 _intoxicated male albino mice**. The results were expressed as mean ± standard error mean.

*In vitro *cell adherence assay reflects the *in vivo *capacity for cellular adherence with treatment by *T. cordifolia *extract in immunocompromised mice. In order to address this fundamental characteristic of macrophages, the cell adhesion property was assayed *in vitro*. Adhesion of cells increased gradually after *T. cordifolia *administration in CCl_4 _intoxicated mice (Figure 2; P = **).

### Effect of *Tinospora cordifolia *on phagocytosis capacity of peritoneal macrophages isolated from CCl_4 _intoxicated male albino mice

**Figure 3:**
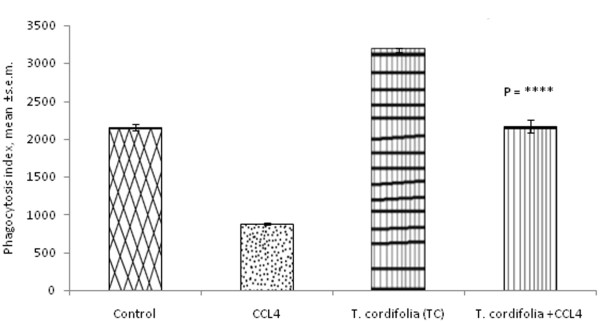
**Effect of *T. cordifolia *on phagocytosis of peritoneal macrophages isolated from CCl_4 _intoxicated male albino mice**. The results were expressed as mean ± standard error mean.

To evaluate the effect of *T. cordifolia *on phagocytosis capacity of peritoneal macrophages, the phagocytosis index was measured. CCl_4 _administration in control mice reduced the phagocytosis index from 2158 ± 39.8 to 886 ± 11.23. But after the administration of *T. cordifolia*, the phagocytosis index of CCl_4 _intoxicated peritoneal macrophages increased up to 2167 ± 86.02. Apart from increasing the phagocytosis index of CCl_4 _intoxicate peritoneal macrophages, *T. cordifolia *treatment also increased the phagocytosis index of control peritoneal macrophages to the level of 3175 ± 32.25 (Figure 3; P = ****).

### Effect of *Tinospora cordifolia *on myeloperoxidase (MPO) enzyme release from peritoneal macrophages isolated from CCl_4 _intoxicated male albino mice

**Figure 4:**
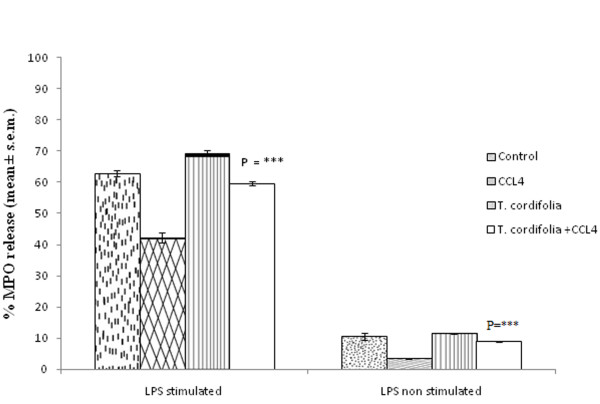
**Effect of *T. cordifolia *on myeloperoxidase release of peritoneal macrophages isolated from CCl_4 _intoxicated male albino mice**. The results were expressed as mean ± standard error mean.

MPO decreases the free radical level in our system. CCl_4 _causes an increase in the level of the free radical CCl_3 _production. The present study was conducted to find whether *T. cordifolia *causes any alteration in the level of free radicals. CCl_4 _intoxication reduces the enzyme release to 42.18 ± 1.69 from 62.81 ± 0.67 as in the case of a control group. However administration of *T. cordifolia *extract in CCl_4 _intoxicated mice sufficed to protect the macrophage and induce 59.5 ± 0.69%. Administration of *T. cordifolia *in control group also showed significant increase of MPO release from target cell up to 69.39 ± 0.89% with LPS stimulation. The same trend was also observed in LPS non-stimulation (Figure 4; P = ****).

### Effect of *Tinospora cordifolia *on nitric oxide (NO) release from peritoneal macrophages isolated from CCl_4 _intoxicated male albino mice

**Figure 5:**
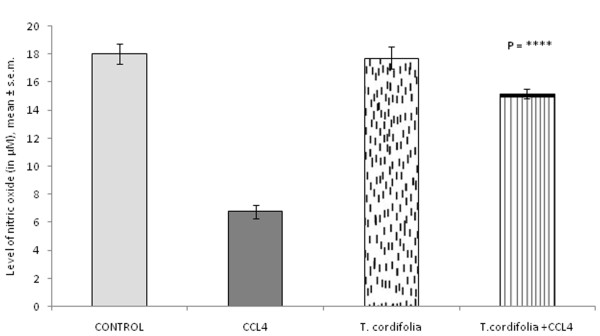
**Effect of *T. cordifolia *on nitric oxide release of peritoneal macrophages isolated from CCl_4 _intoxicated male albino mice**. The results were expressed as mean ± standard error mean.

Nitric oxide plays an important part to kill the microbial pathogen inside the macrophage by forming sodium hypochloride (NaOCl). CCl_4 _intoxication significantly lowered the nitric oxide release to 6.76 ± 0.49 μM from 18.04 ± 0.72 μM as in the case of control group. However *T. cordifolia *administration ameliorated the affect of CCl_4 _in CCl_4 _intoxicated male albino mice (Figure 5; P = ****).

### Effect of *Tinospora cordifolia *on induced DNA fragmentation of peritoneal macrophages by CCl_4_

**Figure 6:**
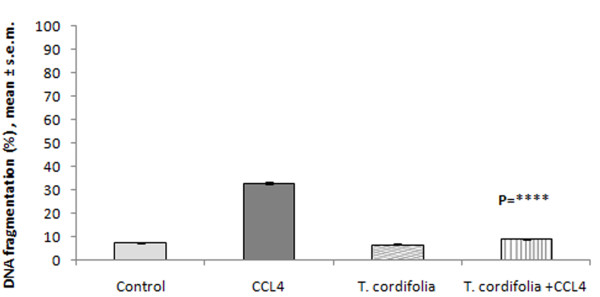
**Effect of *T. cordifolia *on DNA fragmentation of peritoneal macrophages isolated from CCl_4 _intoxicated male albino mice**. The results were expressed as mean ± standard error mean.

In our present study it was found that CCl_4 _induced the DNA fragmentation of peritoneal macrophages by five folds. So, the current work determines whether *T. cordifolia *extract has any ameliorating effect on CCl_4 _induced DNA fragmentation in peritoneal macrophages. The result showed that *T. cordifolia *prevented the DNA fragmentation of CCl_4 _intoxicated peritoneal macrophages (Figure 6; P = ****).

### Effect of *Tinospora cordifolia *on intracellular killing capacity of peritoneal macrophages isolated from CCl_4 _intoxicated male albino mice

**Figure 7:**
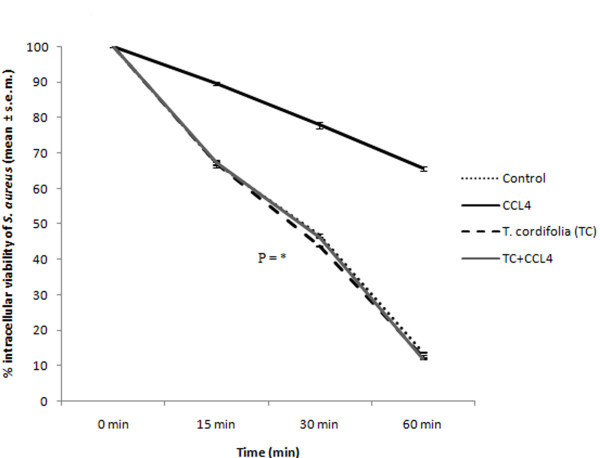
**Effect of *T. cordifolia *on killing capacity of peritoneal macrophages isolated from CCl_4 _intoxicated male albino mice**. The results were expressed as mean ± standard error mean.

Whether *T. cordifolia *administration in CCl_4 _intoxicated mice has any effect on the killing capacity of peritoneal macrophages was a pertinent question. Our results suggested that CCl_4 _intoxication in control mice reduced the intra cellular killing capacity by seven folds but after administration of *T. cordifolia *aqueous extract in CCl_4 _intoxicated mice restored the killing capacity of peritoneal macrophages (Figure 7; P = *).

No acute toxicity was identified on oral administration of the aqueous extract of *Tinospora cordifolia at *a dose of 150 mg/kg body weight.

## Discussion

This study was undertaken to determine the effect of *T. cordifolia *on non specific immune functional activities of peritoneal macrophages in CCl_4 _intoxicated Swiss albino mice. CCl_4 _administration induces hepatic injury and jaundice in a mice model, thus simulating a condition of acute hepatitis and hepatotoxicity. Such conditions can be caused by the excessive breakdown of erythrocytes, impaired liver functions due to viruses or mechanical obstruction of the common bile duct. Such hepatotoxic conditions are almost always accompanied by an immunocompromised state [19] that allows for opportunistic infections which can prove to be fatal. The patient may survive the jaundice but succumb to secondary infections. The present work highlights these indices of impaired immunity associated with hepatic injury and jaundice.

Exposure of organisms to bacterial infection results in the activation of a variety of host defense mechanisms which include phagocytosis, degradative enzyme release and respiratory burst response. The first step in macrophage function is the activation or differentiation of macrophages. However intensive rough surface or almost spherical macrophages can be considered as abnormal macrophages because after getting encountered with the antigen, the macrophages must have perfect surface morphology. In our study, it was found that CCl_4 _intoxication increases this abnormal type of macrophages by several folds. Administration of *T. cordifolia *aqueous extract in these CCl_4 _intoxicated mice renders the macrophages to acquire normal surface morphology so that they function normally. The mechanism involving this protection by *T. cordifolia *extract probably somehow related to the production of monocyte colony stimulating factor or granulocyte-monocyte stimulating factor (M-CSF/GM-CSF).

In order for circulating macrophages to enter inflamed tissue or peripheral lymphoid organs, the cells must adhere to and pass between the endothelial cells lining the walls of blood vessels, a process called extravasation. Endothelial cells express leukocyte-specific cell adhesion molecules (CAMs). Some of these membrane proteins are expressed constitutively; others are expressed only in response to local concentrations of cytokines produced during an inflammatory response. Circulating or peritoneal macrophage bears receptors that bind to CAMs on the vascular endothelium, enabling these cells to extravasate into the tissues. The increase in cell adherence in CCl_4 _intoxicated group after *T. cordifolia *extract treatment might be primarily caused by an increase expression of cell adhesion receptors molecules on the surface of macrophage cells [8].

As is evident from the phagocytic index after CCl_4 _intoxication, it can be suggested that CCl_4 _exposed groups are prone to infections, as they cannot phagocytose efficiently and, as a result, cannot clear out the invading microorganisms, which may lead to a diseased state upon bacterial invasion. However administration of *T. cordifolia *increases the phagocytosis capacity of peritoneal macrophages isolated from CCl_4 _intoxicated mice and this may due to increased number of cells present in the peritoneum as well as alteration of the ability of those that remain functioning normally.

Macrophages secrete lysosomal proteolytic enzymes active at tissue pH that may be important in their ability to kill tumor and bacterial cells. As macrophages mature, there is a progressive rise in lysosomes and hydrolytic content. Continuous CCl_4 _treatment may also lead to immaturation of macrophages *in vivo*, and it can be assumed that due to this exposure they have lesser lysosomal content and hence are less capable of secreting myeloperoxidase (MPO) and nitric oxide (NO). Due to this lesser lysosomal enzyme, these CCl_4 _intoxicated peritoneal macrophages are not able to kill the intracellular *Staphylococcus aureus *and this was evident from our finding increased viability of *Staphylococcus aureus *in CCl-_4 _treated group. The continuous CCl_4 _treatment also increased the DNA fragmentation, which is the indication that the cells were undergoing death and probably due to this they have less functionality. However, treatment by *T. cordifolia *extract during CCl_4 _intoxication may somehow alter the maturation of macrophages as well as prevent the DNA fragmentation, so that they regain their lysosomal hydrolytic content and release more MPO and NO following activation, thus regaining their intracellular killing capacity.

## Conclusion

From our results it can be suggested that the aqueous fraction of *T. cordifolia *stem parts would be effective in ameliorating immunosuppressive effects and prevents pathogenic insults in an immunocompromised state. It can be a good alternative to costly allopathic medicine in boosting the immune functions in intoxicated conditions and can effectively complement allopathic medicines in diseased state.

## Competing interests

The authors declare that they have no competing interests.

## Authors' contributions

MS conceived the study, participated in its design and coordination as well as drafted the manuscript. GDS participated in the design of the study. BC carried out the immunoassays and performed the statistical analysis. All authors read and approved the final manuscript.

## Pre-publication history

The pre-publication history for this paper can be accessed here:

http://www.biomedcentral.com/1472-6882/11/102/prepub
